# Supplementary material for: Dynamic transcriptomic profiles of zebrafish gills in response to zinc supplementation
Source: BMC Genomics. 2010 Oct 11;11:553. doi: 10.1186/1471-2164-11-553 (PMC3091702; doi:10.1186/1471-2164-11-553)
Supplement: Additional file 2 — Interactive Direct Interaction Network representing the molecular interactions between zinc, copper, iron, calcium and proteins encoded by transcripts changed by zinc supplementation. Mini web-site containing index.html and hyperlinked pages in subdirectory describing a Direct Interaction Network automatically generated based on curated interactions contained within the proprietary PathwayArchitect database. Ovals represent proteins and the circles symbolize metal ions. Objects are coloured by their abundance in zebrafish at the time-point they were significantly different from the control is a scale from -4 fold (dark green) to +4 fold (dark red). Where significant differences were found at more than one time-point, the colour overlay shows expression at the first instance. Dark blue squares denote 'binding', and light blue squares 'expression'; green squares stand for 'regulation', green diamonds for 'metabolism', and green circles for 'promoter binding'. Arrow heads indicate directionality of the interaction where annotated. All nodes and edges can be further interrogated by selecting the relative area of the image. [file 1471-2164-11-553-S2.zip › PathwayArchitect Zn xs DIN/400191.html]

# EXPRESSION:

|  |  |
| --- | --- |
| Type | EXPRESSION |
| Effect | None |


---

|  |  |
| --- | --- |
| Score | 0 |


---

|  |  |
| --- | --- |
| Reference Count | 22 |


---

|  |  |
| --- | --- |
| Mechanism | Unknown |


---

|  |  |
| --- | --- |
| Reference:0 || Sentence | "This suggests that factor AP1 (fos/jun) could mediate the up-regulation of collagenase expression in electroporated HDF and HSMC." |
| PMID | 8439282 |
| Year | 1993 |
| Species | Human |
| Journal | Biochem J |
| RefScore | 1 |
| Source | PArchNLP |
  |
|  |  |


---

|  |  |
| --- | --- |
 Reference:1 || Sentence | "Because inflammatory cytokines, e.g., interleukin-1 and tumor necrosis factor-alpha, are considered to activate collagenase gene expression through activation of the transcription factor activator protein-1, we examined whether the water-soluble gold compound aurothiomalate (AuTM) influenced collagenase gene expression, using phorbol ester-treated human fibroblasts." |
| PMID | 7808428 |
| Year | 1994 |
| Species | Human |
| Journal | Mol Pharmacol |
| RefScore | 1 |
| Source | PArchNLP |
  ||  |  |


---

|  |  |
| --- | --- |
 Reference:2 || Sentence | "Activation of the c-fos and c-jun genes is directly involved in expression of metalloproteinases such as collagenase and stromelysin, suggesting that crystal-mediated activation of these genes is directly involved in pathogenesis." |
| PMID | 10823243 |
| Year | 2000 |
| Species | Human |
| Journal | Cell Mol Life Sci |
| RefScore | 0 |
| Source | PArchNLP |
  ||  |  |


---

|  |  |
| --- | --- |
 Reference:3 || Sentence | "Both elements bind factors different in modification or/and constitution from AP-1, the heterodimeric transcription factor composed of c-Fos and c-Jun that controls the activity of the UV and phorbol ester response element (-72 TGAGTCA-66) of the human collagenase gene." |
| PMID | 1561239 |
| Year | 1992 |
| Species | Human |
| Journal | Photochem Photobiol |
| RefScore | 2 |
| Source | PArchNLP |
  ||  |  |


---

|  |  |
| --- | --- |
 Reference:4 || Sentence | "Reduced AP-1 binding activity is associated with reduced expression of the AP-1-dependent gene, collagenase, in old cells treated with DNA damaging agents." |
| PMID | 7790398 |
| Year | 1995 |
| Species | Human |
| Journal | J Cell Physiol |
| RefScore | 0 |
| Source | PArchNLP |
  ||  |  |


---

|  |  |
| --- | --- |
 Reference:5 || Sentence | "These data suggest that collagenase expression is upregulated at the transcriptional level by endogenous activation of DNA binding of AP-1 in CL fibroblasts [corrected]." |
| PMID | 8617996 |
| Year | 1996 |
| Species | Human |
| Journal | J Invest Dermatol |
| RefScore | 1 |
| Source | PArchNLP |
  ||  |  |


---

|  |  |
| --- | --- |
 Reference:6 || Sentence | "Finally, glucocorticoid hormones repress transcription of the collagenase gene by the interaction of glucocorticoid receptors with the AP-1 proteins, Fos and Jun." |
| PMID | 8959374 |
| Year | 1996 |
| Species | Human |
| Journal | Crit Rev Eukaryot Gene Expr |
| RefScore | 1 |
| Source | PArchNLP |
  ||  |  |


---

|  |  |
| --- | --- |
 Reference:7 || Sentence | "Furthermore, VES treatments resulted in increased AP-1 transactivation activity, as measured with an AP-1 promoter/luciferase reporter construct and by the measurement of increased mRNA expression of the AP-1-dependent endogenous gene collagenase." |
| PMID | 9726817 |
| Year | 1998 |
| Species | Human |
| Journal | Mol Carcinog |
| RefScore | 2 |
| Source | PArchNLP |
  ||  |  |


---

|  |  |
| --- | --- |
 Reference:8 || Sentence | "It has been established that the collagenase gene can be activated by c-Jun and c-Fos and that the transcriptional coactivator p300 is involved in the activation." |
| PMID | 12588998 |
| Year | 2003 |
| Species | Human |
| Journal | Mol Cell Biol |
| RefScore | 1 |
| Source | PArchNLP |
  ||  |  |


---

|  |  |
| --- | --- |
 Reference:9 || Sentence | "In the IMR-90 and MG-63 cells, EGF stimulated the transcription of the c-fos and c-jun genes encoding the transcriptional factors which interact directly with the promoter region of the human collagenase gene." |
| PMID | 1648584 |
| Year | 1991 |
| Species | Human |
| Journal | Kokubyo Gakkai Zasshi |
| RefScore | 2 |
| Source | PArchNLP |
  ||  |  |


---

|  |  |
| --- | --- |
 Reference:10 || Sentence | "Overexpression of JunB in HT-1080 cells enhanced collagenase promoter activity by 10-fold, and OA augmented trans-activation of collagenase promoter by c-Jun and JunB." |
| PMID | 7848922 |
| Year | 1994 |
| Species | Human |
| Journal | Cell Growth Differ |
| RefScore | 0 |
| Source | PArchNLP |
  ||  |  |


---

|  |  |
| --- | --- |
 Reference:11 || Sentence | "In addition, regardless of the butyryl group, 4-BPRE actively suppressed c-Jun transcriptional activity, which may result in reduced expression of matrix metalloproteinases (MMP-1 and MMP-2), and effectively inhibited HCT116 cell invasion into Matrigel." |
| PMID | 14767528 |
| Year | 2004 |
| Species | Human |
| Journal | Oncol Rep |
| RefScore | 1 |
| Source | PArchNLP |
  ||  |  |


---

|  |  |
| --- | --- |
 Reference:12 || Sentence | "We show that the AP-1 consensus sequence in the collagenase promoter is required for the induction of collagenase gene expression by okadaic acid, even though sequences upstream of the AP-1 consensus site have an additive effect." |
| PMID | 1966042 |
| Year | 1990 |
| Species | Human |
| Journal | Cell Regul |
| RefScore | 2 |
| Source | PArchNLP |
  ||  |  |


---

|  |  |
| --- | --- |
 Reference:13 || Sentence | "We used the TNF-alpha monoclonal antibody and c-Jun N-terminal kinase (JNK) inhibitor, SP600125, to investigate the cyclical stretch-induced expression of MMP-14 and -2 in cultured HUVECs." |
| PMID | 12909329 |
| Year | 2003 |
| Species | Human |
| Journal | Cardiovasc Res |
| RefScore | 1 |
| Source | PArchNLP |
  ||  |  |


---

|  |  |
| --- | --- |
 Reference:14 || Sentence | "We used wild-type and c-jun null primary and TAg-immortalized mouse embryonic fibroblasts (mEFs) to investigate the importance of c-jun for the regulation of this activity, and observed that c-jun is essential for MMP-2 expression in immortalized but not primary mEFs." |
| PMID | 11414701 |
| Year | 2001 |
| Species | Mouse |
| Journal | Biochem Biophys Res Commun |
| RefScore | 1 |
| Source | PArchNLP |
  ||  |  |


---

|  |  |
| --- | --- |
 Reference:15 || Sentence | "The aim of this study was to investigate whether IL-1beta stimulates the expression of the activator protein 1 (AP-1) transcription factor and, consequently, if the AP-1 transcription factor participates in the regulation of collagenase gene expression in human gingival fibroblast cells." |
| PMID | 11052811 |
| Year | 2000 |
| Species | Human |
| Journal | Cytokine |
| RefScore | 2 |
| Source | PArchNLP |
  ||  |  |


---

|  |  |
| --- | --- |
 Reference:16 || Sentence | "The transcription factor AP1 which regulates expression of collagenase in response to various extracellular signals is a multimeric complex composed of members of the Jun- and Fos families." |
| PMID | 1336108 |
| Year | 1992 |
| Species | Human |
| Journal | Matrix Suppl |
| RefScore | 2 |
| Source | PArchNLP |
  ||  |  |


---

|  |  |
| --- | --- |
 Reference:17 || Sentence | "DNAase I protection analysis indicates that genetically defined enhancer elements in hMT IIA, SV40, and the human collagenase gene contain high-affinity AP-1-binding sites, each with a conserved recognition motif, TGACTCA." |
| PMID | 3034433 |
| Year | 1987 |
| Species | Human |
| Journal | Cell |
| RefScore | 1 |
| Source | PArchNLP |
  ||  |  |


---

|  |  |
| --- | --- |
 Reference:18 || Sentence | "In the dermis and epidermis, AP-1 induces expression of matrix metalloproteinases collagenase, 92 kDa gelatinase, and stromelysin, which degrade collagen and other proteins that comprise the dermal extracellular matrix." |
| PMID | 9732061 |
| Year | 1998 |
| Species | Human |
| Journal | J Investig Dermatol Symp Proc |
| RefScore | 1 |
| Source | PArchNLP |
  ||  |  |


---

|  |  |
| --- | --- |
 Reference:19 || Sentence | "Upregulation of collagenase gene expression by IL-1 is known to require the transactivators Fos and Jun." |
| PMID | 9513043 |
| Year | 1998 |
| Species | Human |
| Journal | J Cell Biochem |
| RefScore | 2 |
| Source | PArchNLP |
  ||  |  |


---

|  |  |
| --- | --- |
 Reference:20 || Sentence | "Since FOS and JUN proteins form a transacting activator (AP-1) for expression of collagenase and stromelysin genes, PC may block the synthesis of both enzymes by inhibiting the transcription of c-fos and c-jun." |
| PMID | 8608166 |
| Year | 1996 |
| Species | Human |
| Journal | Biochim Biophys Acta |
| RefScore | 1 |
| Source | PArchNLP |
  ||  |  |


---

|  |  |
| --- | --- |
 Reference:21 || Sentence | "Transcription of a collagenase promoter/reporter gene was induced in HepG2 hepatoma cells by expression of c-Fos and c-Jun, administration of PMA ("TPA") or by expression of a truncated form of MEK (mitogen-activated/extracellular-signal-regulated kinase kinase) kinase-1, MEKK1Delta." |
| PMID | 11736649 |
| Year | 2001 |
| Species | Human |
| Journal | Biochem J |
| RefScore | 1 |
| Source | PArchNLP |
  |


---

|  |  |
| --- | --- |
